# 2608. Circulating Respiratory Syncytial Virus Genotypes in Hospitalized Children with Bronchiolitis in the United Arab Emirates

**DOI:** 10.1093/ofid/ofad500.2222

**Published:** 2023-11-27

**Authors:** Mohammed Alsamri, Hassib Narchi, Huda AlDhanhani, Junu George, Timothy Collyns, Klaithem Al Dhaheri, Sania Al-Hamad, Ahmed R Alsuwaidi

**Affiliations:** Tawam Hospital, Al Ain, Abu Dhabi, United Arab Emirates; United Arab Emirates University, Al Ain, Abu Dhabi, United Arab Emirates; Sheikh Khalifa Medical City, Abu Dhabi, Abu Dhabi, United Arab Emirates; United Arab Emirates University, Al Ain, Abu Dhabi, United Arab Emirates; Union71, Al Ain, Abu Dhabi, United Arab Emirates; Tawam Hospital, Al Ain, Abu Dhabi, United Arab Emirates; College of Medicine and Health Sciences, UAEU, Al Ain, Abu Dhabi, United Arab Emirates; United Arab Emirates University, Al Ain, Abu Dhabi, United Arab Emirates

## Abstract

**Background:**

Respiratory syncytial virus (RSV) is a major cause of acute respiratory illness in young children and elderly people worldwide. It has a complex molecular epidemiology with multiple strains cocirculating during a single epidemic. Whole genome sequencing is a valuable tool for monitoring genetic diversity and providing genomic information essential for antiviral and vaccine development. In the United Arab Emirates (UAE), little is known about the RSV circulating genotypes, their evolution, and transmission dynamics. Using next-generation sequencing, this study aims to identify RSV genotypes circulating among young children with bronchiolitis in Al Ain City, UAE.

**Methods:**

This cross-sectional study involved 100 children under two years of age who were hospitalized at a tertiary care facility between April and December 2021 with RSV-positive bronchiolitis. Nasopharyngeal samples were obtained, within 24 hours of hospitalization, for RSV genotyping. MinION nanopore sequencer was used to analyze the whole viral genome. We only sequenced samples with cycle threshold (Ct) < 30 (n=70). After sequencing, 7 samples were not analyzed due to long gaps and small lengths. The remaining 63 samples included 59 RSV-A and 4 RSV-B isolates.

**Results:**

The median age of the studied children was 4.3 months, interquartile range (2.1-10.4), with 56% below the age of 6 months. There was no significant difference in the clinical characteristics of children having RSV-A compared to those infected with RSV-B. Phylogenetic analysis showed that out of the 59 RSV-A sequences, 58 clustered with the NA1 genotype, and 1 sequence clustered with the GA1 genotype. All RSV-B sequences were classified as BA2 genotypes. The amino acid sequence alignment of the G gene revealed that all 58 NA1 genotypes had 24 amino acid duplication regions from 247 to 282 amino acid positions. We also found four substitutional changes at L248I, H258Q, L274P, and P276Q.

**Conclusion:**

Phylogenetic analysis revealed that the subtype A NA1 genotype was the dominant strain among the studied children in Al Ain, UAE during the 2021 season. As vaccine development advances, further sequencing of RSV is needed to better understand viral origin, transmission, and genetic recombination, particularly in under-studied populations.

**Disclosures:**

**All Authors**: No reported disclosures

